# A-liner: linear alignment visualizer for genome comparisons

**DOI:** 10.1093/bioinformatics/btag408

**Published:** 2026-06-25

**Authors:** Miki Okuno, Takeshi Yamamoto, Yoshitoshi Ogura, Takehiko Itoh

**Affiliations:** Division of Microbiology, Department of Infectious Medicine, Kurume University School of Medicine, Kurume, Fukuoka 830-0011, Japan; Division of Microbiology, Department of Infectious Medicine, Kurume University School of Medicine, Kurume, Fukuoka 830-0011, Japan; Division of Microbiology, Department of Infectious Medicine, Kurume University School of Medicine, Kurume, Fukuoka 830-0011, Japan; School of Life Science and Technology, Institute of Science Tokyo, Tokyo 152-8550, Japan

## Abstract

**Summary:**

A-liner is a flexible command-line tool for linear visualization of genome-scale sequence alignments, supporting outputs from multiple aligners and integrated visualization of annotations, highlights, quantitative tracks, and coordinate scales. It is applicable to a wide range of organisms, from bacteria to large eukaryotic genomes, and facilitates efficient generation of publication-ready comparative genome visualizations.

**Availability and Implementation:**

The source code and example output files for a-liner are available in the GitHub repository: https://github.com/mokuno3430/a-liner. A-liner v1.1.0 has been archived on Zenodo at https://doi.org/10.5281/zenodo.19702001.

## 1 Introduction

A primary objective of comparative genome analysis is to identify genotypic variants that underlie phenotypic differences among lineages or individuals. Comparing multiple genome sequences and detecting structural variations represent essential first steps toward achieving this goal. Visualization of sequence alignment results is a powerful approach not only for identifying structural variations but also for facilitating a deeper understanding of genome evolution.

Linear comparisons directly represent sequence alignments between genomes and are therefore widely used in comparative genomics analyses, in contrast to other visualization approaches such as dot-plot and circular diagrams. These tools can be broadly categorized into genome alignment visualization tools and gene cluster visualization tools. Genome alignment visualization tools aim to represent large-scale correspondences between genomic regions, whereas gene cluster visualization tools, including Gene Graphics (Harrison *et al*. 2018), clinker & clustermap.js (Gilchrist and Chooi 2021), and LoVis4u (Egorov and Atkinson 2025), focus on local genomic contexts, such as operons or conserved gene neighborhoods, rather than genome-scale comparisons.

Genome alignment visualization tools can be further divided into graphical user interface (GUI)-based and command-line interface (CLI)-based applications. GUI-based tools, such as Mauve (Darling *et al*. 2004), ACT (Carver *et al*. 2005), GenomeMatcher (Ohtsubo *et al*. 2008), GeneSpy (Garcia *et al*. 2019), GEnView (Ebmeyer *et al*. 2022), and GenoFig (Branger and Leclercq 2024), provide user-friendly interfaces and facilitate interactive exploration. However, they typically require manual operation and are less suited to automated workflows. In contrast, CLI-based tools are generally more suitable for automated and reproducible analyses and are widely used in large-scale genomic studies. Representative tools in this category include microbial genome comparison tools such as Easyfig (Sullivan *et al*. 2011) and genoPlotR (Guy *et al*. 2010), as well as tools designed for larger genomes, such as plotsr (Goel and Schneeberger 2022) and MCscan (Wang *et al*. 2012, Tang *et al*. 2024). These tools, however, differ in their primary focus and design. For example, plotsr is optimized for visualizing structural rearrangements, whereas MCscan focuses on gene-level synteny relationships rather than directly visualizing sequence alignments. In addition, highly flexible frameworks, such as gggenomes (Hackl *et al*. 2024), genoPlotR (Guy *et al*. 2010) and GenomeDiagram (Pritchard *et al*. 2006), enable customized visualization but typically require programming expertise, which may limit their accessibility. To enable a systematic comparison, representative CLI-based genome alignment visualization tools with similar design goals are summarized in Table 1.

**Table 1:** Comparison of representative CLI-based genome alignment visualization tools.

tool	target/Scalability	External alignment source	Feature annotation	Manual preprocessing	Additional features	Reference
GenomeDiagram	Flexible (depends on user implementation)	user-provided	GenBank (via Python objects)	Custom scripting required	Python library	Pritchard *et al.* (2006)
genoPlotR	Prokaryotic genomes and eukaryotic genomic regions at small scale	BLAST, Mauve	GenBank, EMBL	Package-based scripting required	R package	Guy *et al.* (2010)
Easyfig	Prokaryotic genomes	BLAST	GenBank, EMBL	Color setup required	GUI (optional)	Sullivan *et al.* (2011)
MCscan (python version)	Prokaryotic to eukaryotic (genome-scale)	BLAST, LAST, MUMmer (gene similarity)	GFF BED (gene-based)	Multiple file (bed, cds and alignment files) preparation required	Gene-level synteny visualization (no sequence-level alignment display)	Wang *et al.* (2012), Tang *et al.* (2024)
plotsr	Prokaryotic to eukaryotic (genome-scale)	minimap2 + SyRI BEDPE format	GFF	Preprocessing required	structural rearrangements	Goel and Schneeberger (2022)
gggenomes	Prokaryotic genomes and moderate datasets	BLAST PAF format	GenBank, GFF	Package-based scripting required	R package	Hackl *et al.* (2024)
a-liner	Prokaryotic to eukaryotic (genome-scale)	BLAST, MUMmer, minimap2, LASTZ	GenBank, GFF	Sequence configuration file required	Integrated multi-layer visualization	this study

Despite the availability of these tools, several limitations remain. First, many existing tools are designed for specific genome sizes or organism types, limiting their applicability to large and complex genomes. Second, the integration of multiple layers of information is often limited or requires extensive manual customization. Third, a trade-off commonly exists between ease of use and flexibility: tools that allow highly customizable visualizations typically require scripting expertise, whereas simpler tools offer limited configurability. These constraints hinder the generation of flexible, scalable, and reproducible visualizations for diverse comparative genomics applications.

To address these limitations, we developed a-liner, a command-line tool for linear visualization of genome-scale sequence alignments. A-liner is designed to complement existing visualization tools by focusing on the generation of integrated, publication-ready figures within a reproducible workflow. It enables the simultaneous visualization of sequence alignments together with gene annotations, highlighted genomic regions, coordinate scales, and user-defined quantitative tracks. By supporting outputs from multiple alignment tools, a-liner provides a flexible framework for diverse comparative genomics applications across both prokaryotic and eukaryotic genomes. In this study, we present representative examples at different genomic scales to demonstrate its practical utility in comparative genomics. Unlike existing tools that focus on specific aspects, a-liner provides integrated, publication-ready visualizations in a reproducible workflow without additional scripting.

## 2 Implementation

### 2.1 Overview

**Figure 1:**
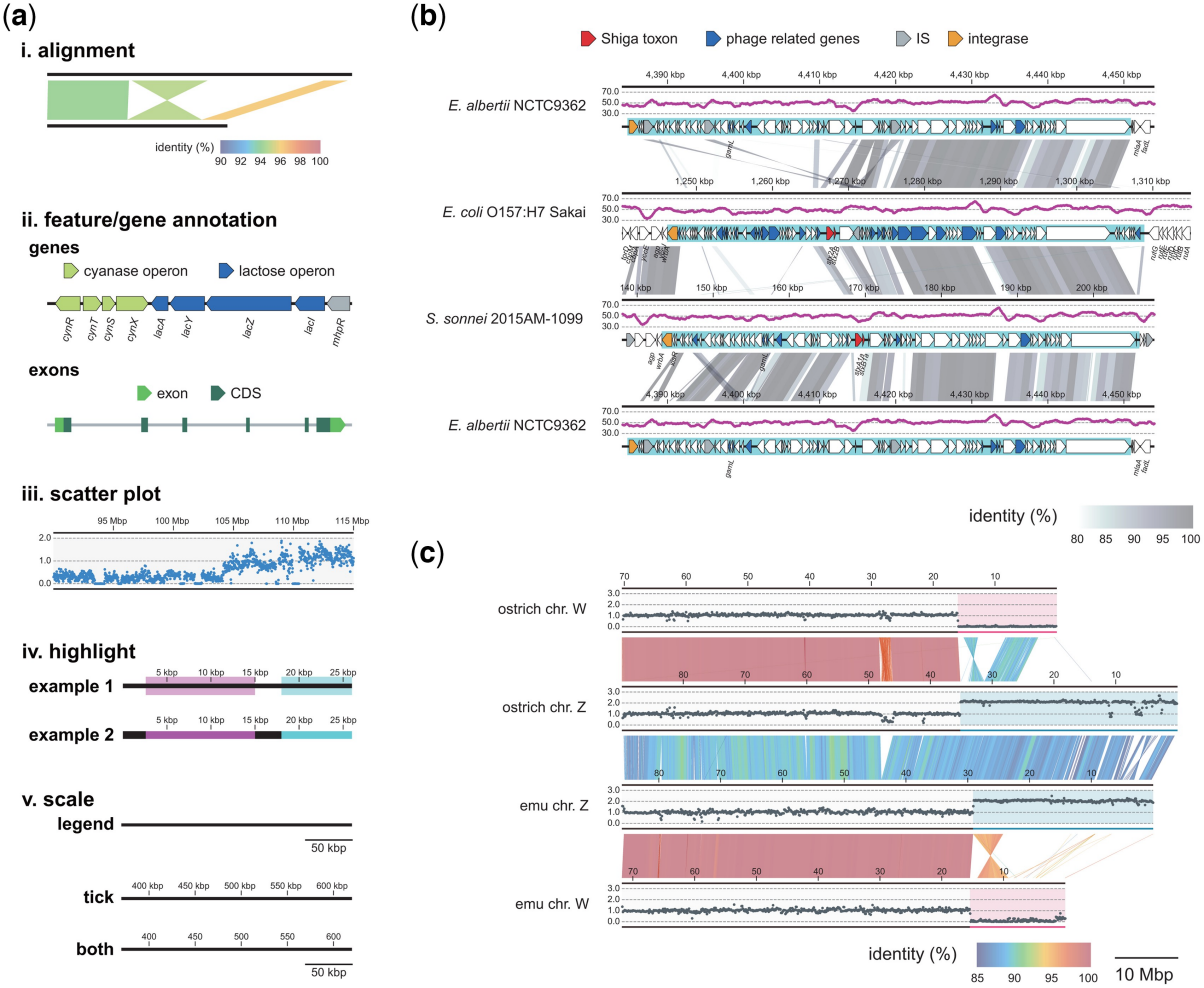
**Overview of a-liner and genome visualization examples**. a. Overview of the visualization components supported by a-liner. The tool enables the display of multiple types of information along genomic sequences, including (i) sequence alignments, (ii) feature or gene annotations, (iii) quantitative tracks such as scatter plots, (iv) highlighted regions or chromosomes, and (v) genomic coordinate scales. These components can be flexibly combined within a single visualization, allowing users to tailor figures to their specific analytical goals. Representative examples of such integrated visualizations are shown in panels b and c. b. Comparison of Stx prophage regions across three *Escherichia–Shigella* strains; *Escherichia albertii* strain NCTC9362, *Escherichia coli* strain Sakai, and *Shigella sonnei* strain 2015AM-1099. Genes are colored according to the legend shown above the figure, and these colors can be customized using the “—feature_color_map” option. GC content (%) is displayed above each sequence track. Alignments were performed using BLASTN, and sequence identity is rendered in grayscale. The “—scale tick” option was used to display genomic coordinates directly on each sequence. Prophage regions are highlighted in light blue using the “—highlight” option. Only the italic formatting of species names was manually adjusted to follow taxonomic conventions. c. Comparison of Z–W sex chromosomes between ostrich and emu. Scatter plots represent the estimated copy number in male individuals. Alignments were calculated using minimap2. Background of the scatter plots were highlighted using the “—sp_highlight” option, and sequence lines were highlighted using the “—highlight” option to indicate putative sex-differentiated regions. The “—scale both” option was applied to display axis ticks along each sequence together with a scale bar.

This tool visualizes sequence alignments with their associated genomic features and annotations in a linear comparison format (Fig. 1a). It focuses on visualization and does not perform sequence alignment itself; instead, it accepts outputs from commonly used sequence alignment tools. In addition to alignment results, the tool also supports standard genome annotation formats, such as GFF and GenBank, as well as user-prepared files in tool-defined formats and generates figures in PDF format. A-liner provides a wide range of options to control sequence layout, color schemes, figure size, and font sizes. For typical use, key visualization parameters are automatically inferred to balance ease of use and reproducibility. All inferred and user-specified parameters are reported during execution, enabling users to reproduce figures under identical settings or to further refine visualizations through detailed customization. The implementation is described below, following the general workflow, from sequence preparation to visualization customization. In addition to command-line usage, a-liner can be invoked programmatically from Python by passing argument lists directly to the execution function. This enables integration into Python-based scripting environments without requiring manual CLI interaction.

### 2.2 Setting up sequences information

Sequence layout information can be specified using either tab-delimited (TSV) or Excel files with a unified format. In this context, a track refers to a horizontal layer in the figure; multiple sequences can be arranged horizontally within the same track, and multiple tracks can be stacked vertically.

Each row represents a sequence and includes columns for track index (0-based, with 0 indicating the bottom track), sequence ID, start position, end position, orientation (+ or –), and display name. Genomic coordinates are specified using a 1-based system. When multiple sequences are assigned to the same track index, they are arranged from left to right according to their order in the file.

### 2.3 Visualization of sequence alignments

A-liner accepts alignment results generated by commonly used sequence alignment tools, including BLASTN (Altschul *et al*. 1990) output in tabular format (—outfmt 6), LASTZ (Harris 2007) output in general tabular format (—format=general), PAF files with CIGAR strings produced by minimap2 (Li 2018) using the -c option, and coordinate tables generated by the show-coords utility in MUMmer (Marçais *et al*. 2018). Alignment results can be filtered based on sequence identity and alignment length using user-defined thresholds. Sequence identity is represented using a colormap, with a rainbow colormap applied by default, and alternative colormaps can be specified via a command-line option. By default, alignments are drawn only between vertically adjacent sequences in the figure; this restriction can be lifted to include alignments between non-adjacent sequences, enabling visualization of more complex relationships across multiple sequences.

### 2.4 Visualization of genome annotations

A-liner supports the visualization of gene structures by reading genome annotation files in GFF3 and GenBank formats. In addition, users can flexibly assign colors to genomic features using a user-defined mapping file (—feature_color_map), which enables automatic coloring based on gene names or functional annotations and generates a corresponding legend in the figure. The mapping file is a simple tab-separated table consisting of legend label, keywords, and color codes, where features whose gene names or descriptions match the specified keywords are automatically colored. Multiple keywords can be specified in the second column by separating them with slashes (/). Users can also individually specify the display color of each gene by providing a GFF3 file in which an additional column containing color codes (e.g. #FF0000) is appended to the standard format. In addition, the tool can read Excel files in which GFF3 formats are pasted, allowing users to conveniently edit gene names and display colors directly within the spreadsheet. By default, features annotated as genes are visualized; however, other feature types, such as CDS, can also be specified for rendering using the—feature option. Unlike approaches that require manual editing of annotation files, this system enables automatic and reproducible feature coloring based on annotation keywords. This design facilitates integration into automated analysis pipelines and ensures consistent visualization.

### 2.5 Highlighting genomic regions

The—highlight option allows users to visually emphasize specific genomic features, such as gene clusters or regions affected by structural variation. Highlighted regions are specified using a tab-delimited file containing sequence IDs, start and end positions, and color specifications. The thickness and transparency of the highlighted regions can be controlled using the—h_thickness and—h_alpha options, respectively. By setting these parameters to their maximum values (thickness = 1 and alpha = 1), the—highlight option allows solid coloring to be applied to user-defined regions or entire sequences, enabling color customization at the level of individual sequences or specific subregions.

### 2.6 Displaying scales

The tool provides three options for displaying coordinate scales within figures. With the default—scale legend option, a scale bar is shown in the lower-right corner of the figure. When—scale tick is specified, coordinate ticks are plotted directly along each sequence. The—scale both option displays both the scale bar and coordinate ticks. When only a specific region of a chromosome or sequence is visualized, the tick option indicates the corresponding genomic coordinates based on the original coordinate system, making the displayed range explicit. Tick intervals are determined automatically, but users can manually specify the interval using the—tick_width option.

### 2.7 Adding scatter plots

The scatter plot functionality enables the simultaneous visualization of structural relationships between sequences and associated quantitative features, such as read depth or SNP density. Scatter plots are displayed above each sequence track by specifying a tab-delimited file containing sequence IDs, genomic positions, and arbitrary numerical values via the—scatter option. Marker shape, color, and size, as well as the *y*-axis range and tick intervals, can be customized using command-line options.

## 3 Results

We present four practical examples of genome visualization using a-liner. All visualizations presented in this study were generated using a-liner v1.1.0. As the first example, we illustrate a comparative analysis of Shiga toxin (Stx)-encoding regions among *Escherichia–Shigella* strains (Fig. 1b). Stx is encoded by chromosomally integrated prophages, and we compared the Stx prophage region of *Escherichia coli* O157: H7 strain Sakai with corresponding regions from two closely related species (Hayashi *et al*. 2001, Lindsey *et al*. 2017, Carter *et al*. 2023). Alignment results and gene annotations were visualized together, with prophage regions highlighted to emphasize structural correspondence. Homologous regions were clearly identified across all strains, allowing differences in prophage conservation across the strains to be intuitively visualized.

Next, we demonstrate the use of a-liner for chromosome-scale comparisons in eukaryotic genomes using the Z–W sex chromosomes of emu and ostrich (Fig. 1c) (Okuno *et al*. 2021, Rhie *et al*. 2021, Yazdi *et al*. 2023). Sequence alignments were visualized together with scatter plots of estimated copy numbers in male (ZZ) individuals. The figure shows boundaries of copy number changes along the sex chromosomes, corresponding to putative breakpoints between Z and W differentiation. Differences in sequence homology and in the positions of inversions within putative non-recombining regions between emu and ostrich are readily observable. This example demonstrates that a-liner enables intuitive interpretation of chromosome-scale differentiation by integrating structural and quantitative genomic features.

To further demonstrate additional functionalities of a-liner, we included examples highlighting exon–intron visualization. At the *Ddx3x* and *Ddx3y* loci in mouse, a-liner clearly displays exon–intron structures together with genomic alignments generated by multiple aligners (BLASTN, LASTZ, minimap2, and MUMmer) (Fig. S1, available as supplementary data at *Bioinformatics* online). Coding sequences and exons are visually distinguished, enabling intuitive interpretation of gene structure and strand orientation even between highly diverged gametologs. Alignment patterns vary depending on the alignment tool and parameter settings used. In this example, exon regions are consistently aligned across all tools, while differences in alignment continuity are observed. For instance, under the parameter settings used here, LASTZ produces more continuous alignments in some regions, whereas other tools tend to produce more fragmented patterns. These observations reflect the characteristics of the alignment tools and are not intended as a systematic performance comparison.

As an example of multi-sequence placement on a single track, we visualized whole-chromosome alignments among *Apodemus sylvaticus*, *Tokudaia tokunoshimensis*, and *Tokudaia osimensis* (Okuno *et al*. 2023) (Fig. S2, available as supplementary data at *Bioinformatics* online). By placing multiple chromosomes from one species on a single track, a-liner enables direct comparison of one genomic region against multiple corresponding regions in another genome. This configuration clearly reveals one-to-multiple relationships, such as chromosome fusion events, demonstrating the effectiveness of multi-sequence placement for comparative genomics.

## 4 Conclusion

A-liner is broadly applicable to genome comparisons across a wide range of organisms and scales, from microbial genomes to chromosome-scale analyses in eukaryotes. It supports both rapid, exploratory visualization and the generation of publication-ready figures within a reproducible workflow. By enabling the integrated visualization of sequence alignments with multiple layers of genomic information, a-liner provides a flexible and practical framework for comparative genomics. Overall, a-liner facilitates efficient analysis and reproducible figure generation tailored to users’ specific analytical goals.

## Supplementary Material

btag408_Supplementary_Data

## Data Availability

The source code and example output files for a-liner are available in the GitHub repository: https://github.com/mokuno3430/a-liner, with an archived version on Zenodo (https://doi.org/10.5281/zenodo.19702001). The supplementary datasets used to generate the figures are also available on Zenodo (https://doi.org/10.5281/zenodo.19695796). Information on the datasets used in this study is provided in the Supplementary Material.

## References

[btag408-B1] Altschul SF , Gish W, Miller W et al Basic local alignment search tool. J Mol Biol 1990;215:403–10. 2231712 10.1016/S0022-2836(05)80360-2

[btag408-B2] Branger M , Leclercq SO. GenoFig: a user-friendly application for the visualization and comparison of genomic regions. Bioinformatics 2024;40:btae372. 38870520 10.1093/bioinformatics/btae372 PMC11199195

[btag408-B3] Carter MQ , Quiñones B, He X et al Genomic and phenotypic characterization of Shiga toxin-producing *Escherichia albertii* strains isolated from wild birds in a major agricultural region in California. Microorganisms 2023;11:2803. 38004814 10.3390/microorganisms11112803 PMC10673567

[btag408-B4] Carver TJ , Rutherford KM, Berriman M et al ACT: the Artemis comparison tool. Bioinformatics 2005;21:3422–3. 15976072 10.1093/bioinformatics/bti553

[btag408-B5] Darling ACE , Mau B, Blattner FR et al Mauve: multiple alignment of conserved genomic sequence with rearrangements. Genome Res 2004;14:1394–403. 15231754 10.1101/gr.2289704 PMC442156

[btag408-B6] Ebmeyer S , Coertze RD, Berglund F et al GEnView: a gene-centric, phylogeny-based comparative genomics pipeline for bacterial genomes and plasmids. Bioinformatics 2022;38:1727–8. 34951622 10.1093/bioinformatics/btab855 PMC8896611

[btag408-B7] Egorov AA , Atkinson GC. LoVis4u: a locus visualization tool for comparative genomics and coverage profiles. NAR Genom Bioinform 2025;7:lqaf009. 40007724 10.1093/nargab/lqaf009 PMC11850299

[btag408-B8] Garcia PS , Jauffrit F, Grangeasse C et al GeneSpy, a user-friendly and flexible genomic context visualizer. Bioinformatics 2019;35:329–31. 29912383 10.1093/bioinformatics/bty459

[btag408-B9] Gilchrist CLM , Chooi YH. Clinker & clustermap.js: automatic generation of gene cluster comparison figures. Bioinformatics 2021;37:2473–5. 33459763 10.1093/bioinformatics/btab007

[btag408-B10] Goel M , Schneeberger K. Plotsr: visualizing structural similarities and rearrangements between multiple genomes. Bioinformatics 2022;38:2922–6. 35561173 10.1093/bioinformatics/btac196 PMC9113368

[btag408-B11] Guy L , Roat Kultima J, Andersson SGE. genoPlotR: comparative gene and genome visualization in R. Bioinformatics 2010;26:2334–5. 20624783 10.1093/bioinformatics/btq413 PMC2935412

[btag408-B12] Hackl T , Ankenbrand M, Adrichem B et al gggenomes: effective and versatile visualizations for comparative genomics. *arXiv preprint arXiv : 2411*.*13556,* 2024, preprint: not peer reviewed.

[btag408-B13] Harris RS. Improved Pairwise Alignment of Genomic DNA. The Pennsylvania State University, 2007.

[btag408-B14] Harrison KJ , Crécy-Lagard V, de Zallot R et al Gene graphics: a genomic neighborhood data visualization web application. Bioinformatics 2018;34:1406–8. 29228171 10.1093/bioinformatics/btx793 PMC5905594

[btag408-B15] Hayashi T , Makino K, Ohnishi M et al Complete genome sequence of enterohemorrhagic *Escherichia coli* O157: h 7 and genomic comparison with a laboratory strain K-12. DNA Res 2001;8:11–22. 11258796 10.1093/dnares/8.1.11

[btag408-B16] Lindsey RL , Batra D, Rowe L et al High-quality draft genome sequences for four drug-resistant or outbreak-associated *Shigella sonnei* strains generated with PacBio sequencing and whole-genome maps. Genome Announc 2017;5. 10.1128/genomea.00906-17 PMC5578855 28860257

[btag408-B17] Li H. Minimap2: pairwise alignment for nucleotide sequences. *Bioinformatics* 2018;34:3094–100. 10.1093/bioinformatics/bty191 29750242 PMC6137996

[btag408-B18] Marçais G , Delcher AL, Phillippy AM et al MUMmer4: a fast and versatile genome alignment system. PLoS Comput Biol 2018;14:e1005944. 29373581 10.1371/journal.pcbi.1005944 PMC5802927

[btag408-B19] Ohtsubo Y , Ikeda-Ohtsubo W, Nagata Y et al GenomeMatcher: a graphical user interface for DNA sequence comparison. BMC Bioinformatics 2008;9:376. 18793444 10.1186/1471-2105-9-376 PMC2553346

[btag408-B20] Okuno M , Mizushima S, Kuroiwa A et al Analysis of sex chromosome evolution in the clade palaeognathae from phased genome assembly. Genome Biol Evol 2021;13:evab242. 34718546 10.1093/gbe/evab242 PMC8599748

[btag408-B21] Okuno M , Mochimaru Y, Matsuoka K et al Chromosomal-level assembly of *Tokudaia osimensis, Tokudaia tokunoshimensis*, and *Tokudaia muenninki* genomes. Sci Data 2023;10:927. 38129438 10.1038/s41597-023-02845-1 PMC10739956

[btag408-B22] Pritchard L , White JA, Birch PRJ et al GenomeDiagram: a python package for the visualization of large-scale genomic data. Bioinformatics 2006;22:616–7. 16377612 10.1093/bioinformatics/btk021

[btag408-B23] Rhie A , McCarthy SA, Fedrigo O et al Towards complete and error-free genome assemblies of all vertebrate species. Nature 2021;592:737–46. 33911273 10.1038/s41586-021-03451-0 PMC8081667

[btag408-B24] Sullivan MJ , Petty NK, Beatson SA. Easyfig: a genome comparison visualizer. Bioinformatics 2011;27:1009–10. 21278367 10.1093/bioinformatics/btr039 PMC3065679

[btag408-B25] Tang H , Krishnakumar V, Zeng X et al JCVI: a versatile toolkit for comparative genomics analysis. IMeta 2024;3:e211. 39135687 10.1002/imt2.211 PMC11316928

[btag408-B26] Wang Y , Tang H, DeBarry JD et al MCScanX: a toolkit for detection and evolutionary analysis of gene synteny and collinearity. Nucleic Acids Res 2012;40:e49. 22217600 10.1093/nar/gkr1293 PMC3326336

[btag408-B27] Yazdi HP , Olito C, Kawakami T et al The evolutionary maintenance of ancient recombining sex chromosomes in the ostrich. PLoS Genet 2023;19:e1010801. 37390104 10.1371/journal.pgen.1010801 PMC10343094

