# Supplementary material for: A-liner: linear alignment visualizer for genome comparisons
Source: Bioinformatics. 2026 Jun 25;42(6):btag408. doi: 10.1093/bioinformatics/btag408 (PMC13318494; doi:10.1093/bioinformatics/btag408)
Supplement: btag408_Supplementary_Data [file btag408_supplementary_data.pdf]

Supplementary Data

A-liner: linear alignment visualizer for genome comparisons

Data availability and source datasets

The source code and example output files for a-liner are available from the GitHub repository:
https://github.com/mokuno3430/a-liner
A-liner v1.1.0 has been archived on Zenodo (DOI: 10.5281/zenodo.19702001).
The supplementary datasets are available on Zenodo (DOI: 10.5281/zenodo.19695796).

The following publicly available datasets, all available through the NCBI databases, were used in this study.

Figure 1b
Comparison of Stx prophage regions among three *Escherichia–Shigella* strains.

- *Escherichia albertii* strain NCTC9362 (RefSeq: GCF_003864095.1)
- *Escherichia coli* O157:H7 strain Sakai (RefSeq: GCF_000008865.2)
- *Shigella sonnei* strain 2015AM-1099 (RefSeq: GCF_002142635.1)

Figure 1c
Comparison of Z–W sex chromosomes between ostrich and emu.
Reference genomes:

- *Struthio camelus* isolate bStrCam1 (RefSeq: GCF_040807025.1)
- *Dromaius novaehollandiae* isolate bDroNov1 (RefSeq: GCF_036370855.1)

Whole-genome sequencing datasets:

- *S. camelus* male individual (SRA: SRR24085069)
- *D. novaehollandiae* male individual (SRA: DRR233937)

Figure S1
Exon–intron structures and comparative genomic alignments at the *Ddx3x* and *Ddx3y* loci in mouse.

- *Mus musculus* chromosome X (RefSeq: NC_000086.8)
- *Mus musculus* chromosome Y (RefSeq: NC_000087.8)

Figure S2
Chromosome-scale comparison among *Apodemus sylvaticus*, *Tokudaia tokunoshimensis*, and *Tokudaia osimensis*.

- *A. sylvaticus* (RefSeq: GCF_947179515.1)
- *T. tokunoshimensis* (GenBank: GCA_036184795.1)
- *T. osimensis* (WGS master accession: BTPL01000000)

Supplementary Figures

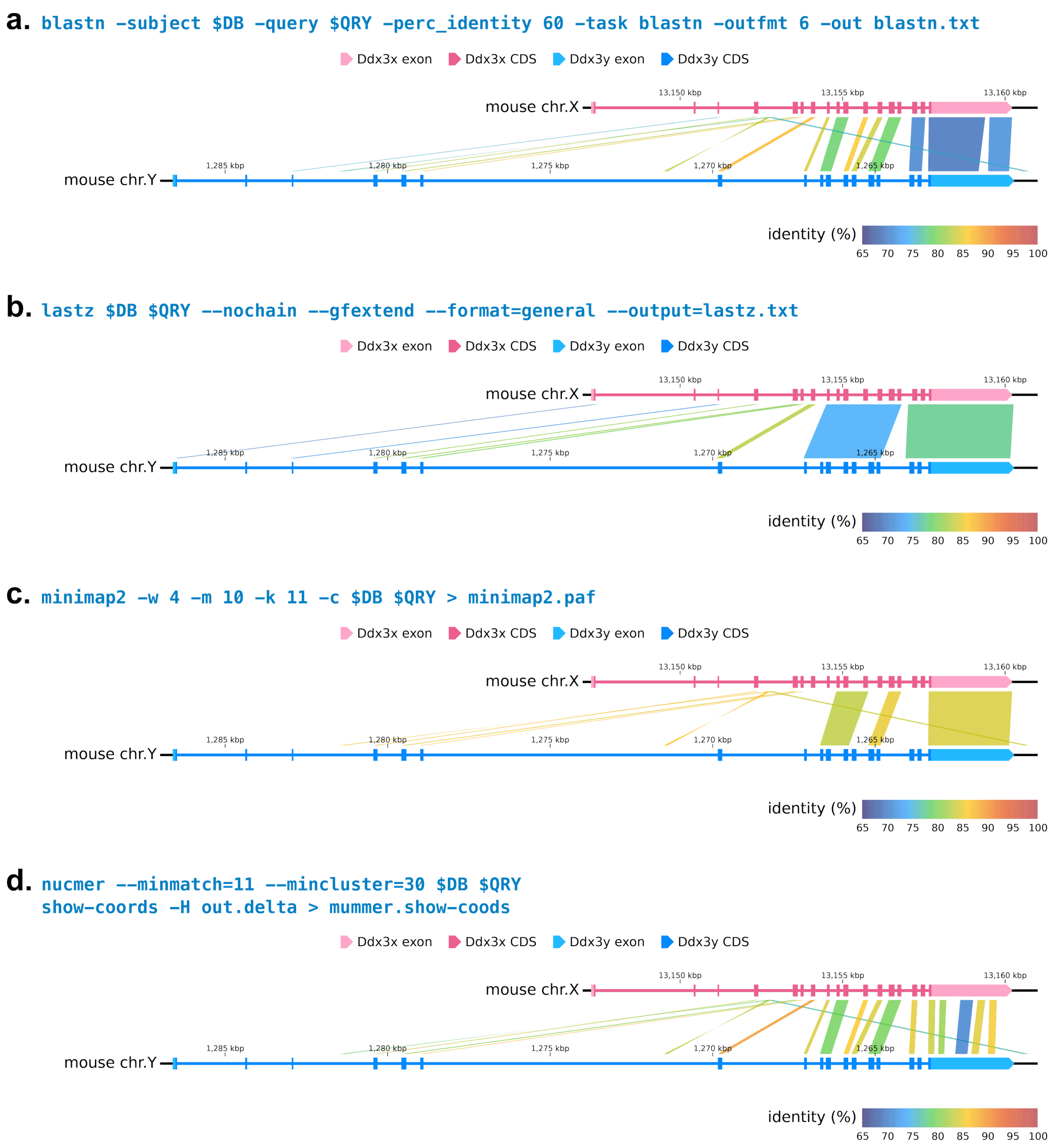

Figure S1: Exon–intron structures and comparative genomic alignments at the *Ddx3x* and *Ddx3y* loci in mouse. Panels (a–d) show alignments generated using BLASTN (a), LASTZ (b), minimap2 (c), and MUMmer (d), respectively, and visualized with a-liner. *Ddx3x* and *Ddx3y* are gametologous genes located on the X and Y chromosomes and exhibit substantial sequence divergence. Exon–intron structures are displayed using the --feature CDS exon option. Coding sequences (CDS) and exons are distinguished by color using --feature_color_map, allowing clear identification of coding regions and UTRs. In a-liner, exon structures can be visualized using the --feature exon option. The terminal exon of each mRNA is represented as an arrow-shaped box indicating strand orientation, while the remaining exons are shown as rectangular boxes.

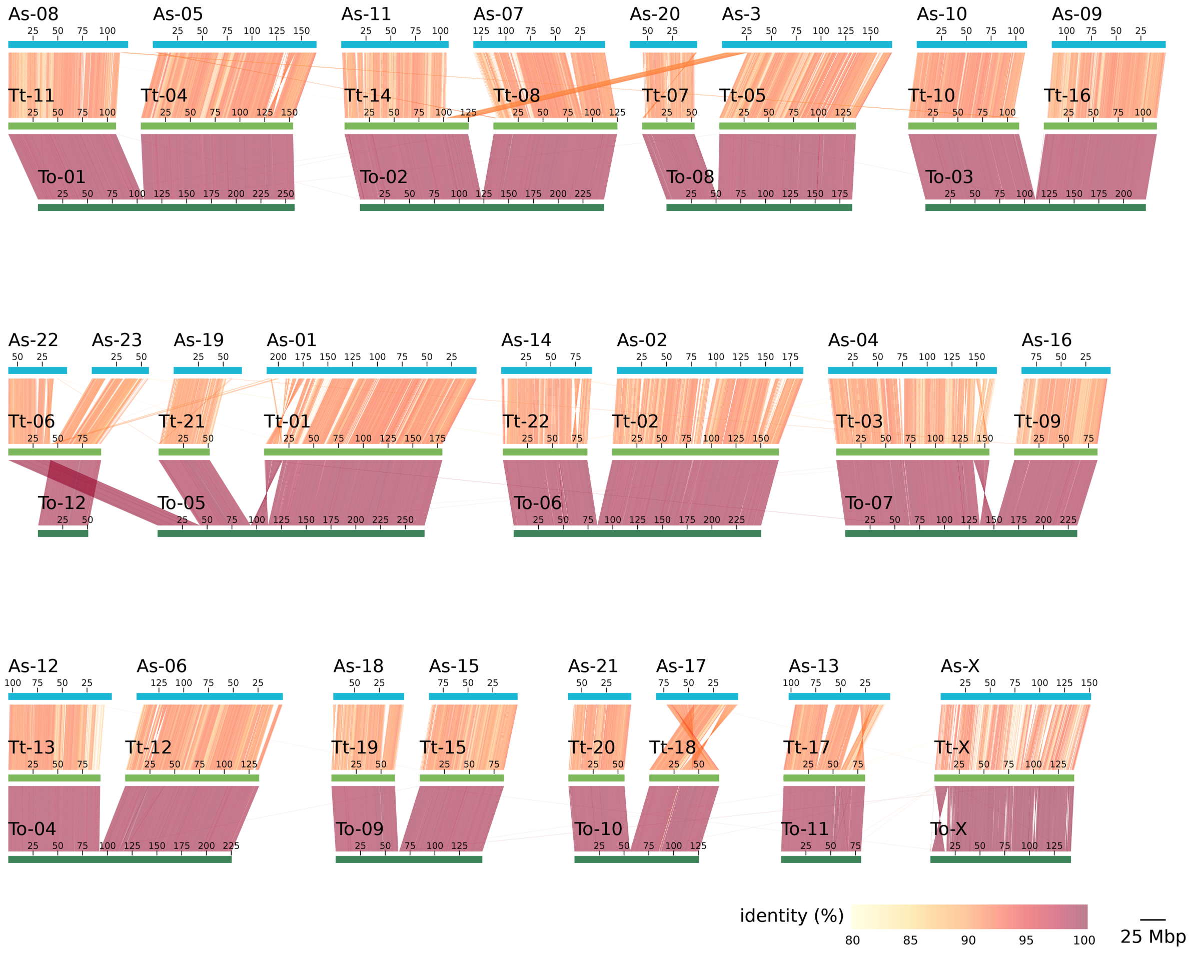

Figure S2: Chromosome-to-chromosome alignment demonstrating multi-sequence placement on a single track. Whole-chromosome alignments among *Apodemus sylvaticus*, *Tokudaia tokunoshimensis*, and *Tokudaia osimensis* are shown. *T. tokunoshimensis* and *T. osimensis* are closely related, while *A. sylvaticus* serves as an outgroup. Autosomes and X chromosomes are included in the analysis. Alignments were generated using minimap2. Chromosomes are labeled according to species and chromosome number: "As-" indicates *A. sylvaticus*, "Tt-" indicates *T. tokunoshimensis*, and "To-" indicates *T. osimensis*, followed by the chromosome number (e.g., As-01). Multiple chromosomes from one species are placed on the same tracks, enabling direct comparison with chromosomes from another species. This visualization clearly reveals one-to-multiple relationships among chromosomes, including fusion events, and illustrates the capability of a-liner to display multiple sequences on a single track.
